# Innervation of the cricothyroid muscle by extralaryngeal branches of the recurrent laryngeal nerve

**DOI:** 10.1002/lary.25691

**Published:** 2015-10-28

**Authors:** Akira Miyauchi, Hiroo Masuoka, Ayako Nakayama, Takuya Higashiyama

**Affiliations:** ^1^ Department of SurgeryKuma HospitalKobeJapan; ^2^ Department of Head and Neck SurgeryKuma HospitalKobeJapan

**Keywords:** Recurrent laryngeal nerve, extralaryngeal branch, cricothyroid muscle, innervation, voice change

## Abstract

**Objectives/Hypothesis:**

A major concern in thyroid surgery is possible changes in the patient's voice due to dysfunction of the laryngeal muscles. The classical understanding of the anatomy is that the cricothyroid muscle (CTM) is innervated solely by the external branch of the superior laryngeal nerve (EBSLN), and the endolaryngeal muscles are covered only by the recurrent laryngeal nerve (RLN). Meticulous anatomical studies found communication between these nerves. Recent neurophysiological studies revealed cross‐innervations among these nerve–muscle sets. Here, we report innervation of the CTM by extralaryngeal branches of the RLN.

**Study Design:**

Clinical observation during thyroid surgery at a hospital center for thyroid diseases.

**Methods:**

During thyroid cancer surgeries, we encountered four adult Japanese patients who had an extralaryngeal branch of the RLN, the electrical stimulation of which showed contraction of the CTM. The EBSLN and RLN were electrically stimulated. Responses were evaluated by visual observation of contraction of the CTM and palpable laryngeal twitch of the endolaryngeal muscles. Electromyographic studies were also performed in two patients.

**Results:**

Five of the seven RLNs examined showed contraction of the CTM on stimulation. Four of these five RLNs had an extralaryngeal branch that showed contraction of the CTM on stimulation. Stimulation of the RLN proximal to the branch yielded contraction of the CTM and laryngeal twitch, whereas stimulation of the RLN distal to the branch yielded only laryngeal twitch.

**Conclusions:**

Extralaryngeal branches of the RLN innervated the CTM in four patients. This phenomenon might influence voice changes following thyroid surgery.

**Level of Evidence:**

4. *Laryngoscope*, 126:1157–1162, 2016

## INTRODUCTION

One of the major concerns in thyroid surgery is the possible change in the patient's voice due to dysfunction of the laryngeal muscles. The laryngeal muscles are closely involved in phonation and are innervated by the recurrent laryngeal nerve (RLN) and the external branch of the superior laryngeal nerve (EBSLN). The classical understanding of the anatomy is that the EBSLN innervates the cricothyroid muscle (CTM), and the RLN covers the endolaryngeal muscles other than the CTM.^1^ The contraction of the CTM tilts the thyroid cartilage against the cricoid cartilage. This motion increases the length and tension of the vocal folds, making a high tone and strong voice.^1^

Meticulous anatomical studies of cadaver larynges revealed terminal branches of the EBSLN reaching the thyroarytenoid muscle and communicating with the branches of the RLN in the larynx.^2^, ^3^, ^4^ These branches are called “human communicating nerves.”^5^ Recent intraoperative nerve monitoring studies with a tracheal tube with surface electrodes revealed the activation of the endolaryngeal muscles when the EBSLN was electrically stimulated, in 70% to 80% of patients.^6^ This phenomenon is used in one of the intraoperative methods for monitoring the EBSLN.^6^ Martin‐Oviedo et al. found functional innervation in the inverse direction, from the RLN to the CTM, in seven patients.^7^ We recently found and reported that at least 39% and possibly 73% of 70 RLNs examined electrophysiologically during thyroid surgery showed this inverse innervation to the CTM.^8^ To the best of our knowledge, however, innervation of the CTM by extralaryngeal branches of the RLN has not been reported. Here, we describe inverse innervation of the CTM by extralaryngeal branches of the RLN that might influence voice changes following thyroid surgery.

## MATERIALS AND METHODS

Between December 2012 and February 2015, we encountered four Japanese papillary thyroid carcinoma patients who showed an extralaryngeal branch of the RLN during thyroid surgery; the branches showed contraction of the CTM on electrical stimulation. The patients, two women and two men, were aged 42 to 62 years. Three of the patients underwent a total thyroidectomy and central node dissection, and the remaining patient underwent a left hemithyroidectomy and ipsilateral paratracheal node dissection. During these surgeries, the EBSLN, the vagus, and the RLN were electrically stimulated. Responses were evaluated by visual observation of contraction of the CTM, that is, CTM twitch, for the EBSLN^9^ and by laryngeal twitch of the endolaryngeal muscles for the RLN.^10^

Electromyographic studies were also performed in two of the patients, using a tracheal tube with surface electrodes^11^ and a pair of needle electrodes inserted into the pars recta of the CTM. We used the NIM 3.0 system (Medtronic, Jacksonville, FL) and stimulated the nerves using a monopolar probe and the interrupted stimulation technique at 1 to 2 mA, 100‐millisecond impulse duration, and 4‐Hz frequency. Laryngoscopy was performed preoperatively and postoperatively with a flexible laryngoscope.

All of the patients underwent a phonation function test preoperatively and postoperatively using the Phonation Analyzer PA‐1000 (Minato Medical Science, Osaka, Japan).^12^ The patient's voice pitch, the voice level of his or her fundamental voice and high‐tone voice, and the maximum phonation time were recorded with the analyzer. The ethical committee of Kuma Hospital approved the present study, and all patients gave informed consent for the electromyographic studies.

## RESULTS

### Patient 1

**Figure 1:**
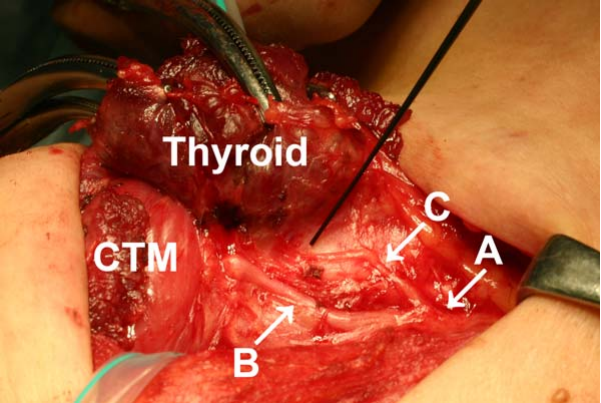
The right recurrent laryngeal nerve (RLN) and its branch in patient 1, a 42‐year‐old woman. Electrical stimulation of the RLN proximal to the branch (A) evoked a twitch of the cricothyroid muscle (CTM) as well as a laryngeal twitch, whereas stimulation of the RLN distal to the branch (B) caused a laryngeal twitch only. Stimulation of the branch (C) yielded a CTM twitch without a laryngeal twitch. The branch ran cranially, penetrated Berry's ligament, and then ran anteriorly toward the origin of the CTM, where it became untraceable. Head is to the left.

A 42‐year‐old woman underwent a total thyroidectomy with central node dissection. The right EBSLN was of Cernea classification type 2B,^13^ which was identified with electrical stimulation and visual identification of the CTM twitch. During the process of resection of the right lobe, we found a branch originating from the right RLN approximately 3 cm caudal to the laryngeal entry point (Fig. 1). Electrical stimulation of the branch (C in Fig. 1) evoked clear visual contraction of the right CTM. Electrical stimulation of the RLN proximal to the branch (A in Fig. 1) also yielded a CTM twitch as well as a laryngeal twitch; however, stimulation of the RLN distal to the branch (B in Fig. 1) showed the laryngeal twitch only (see Supporting Video 1 in the online version of this article).

The branch ran cranially on the surface of the trachea, penetrated Berry's ligament, and then ran anteriorly, reaching near the head of the right CTM, where it became untraceable. Stimulation along the whole course of the branch evoked a CTM twitch, whereas laryngeal twitch was negative. During the dissection of the nerve, this response disappeared, probably due to neural paresis.

These findings showed that the contraction of the CTM was not secondary to the contraction of the endolaryngeal muscles, and that the branch of the RLN innervated the CTM in this patient. The left EBSLN was of Cernea classification type 1. Stimulation of the left RLN did not show CTM twitch, although laryngeal twitch was clearly palpated. Postoperatively, the patient did not exhibit or complain of any voice problems.

### Patient 2

**Figure 2:**
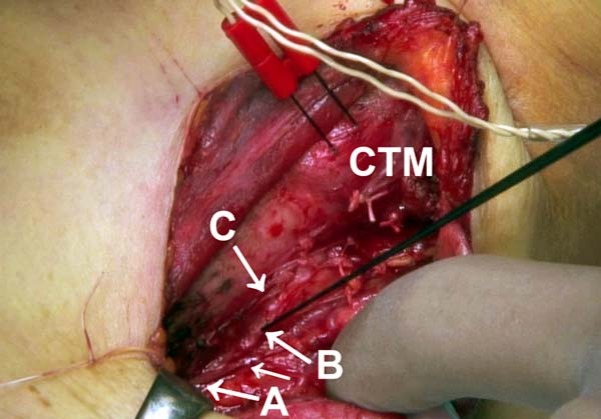
The left recurrent laryngeal nerve (RLN), its anterior branch (C), and its posterior branch (short arrow) in patient 2, a 54‐year‐old woman. A pair of electrodes was inserted into the left cricothyroid muscle (CTM). A monopolar probe stimulated the anterior branch, yielding contraction of the CTM on electromyography without contraction of the glottis muscles. Stimulation of the RLN proximal to the branch (A) evoked contraction of the glottis muscle and the CTM on electromyographic studies, whereas stimulation of the RLN distal to the branch (B) yielded contraction of the glottis muscle only.

A 54‐year‐old woman underwent a left hemithyroidectomy and paratracheal node dissection for a papillary microcarcinoma of the thyroid that was located close to the left RLN. A tracheal tube with surface electrodes for intraoperative nerve monitoring was placed between her vocal folds. A pair of needle electrodes was inserted into the pars recta of the left CTM before the EBSLN, the vagus, and the RLN were stimulated. During the surgery, we found two branches originating from her left RLN approximately 4 cm caudal to the laryngeal entry point. One of the branches ran anteriorly and cranially along the RLN, and the other ran dorsally to the esophagus (Fig. 2).

**Figure 3:**
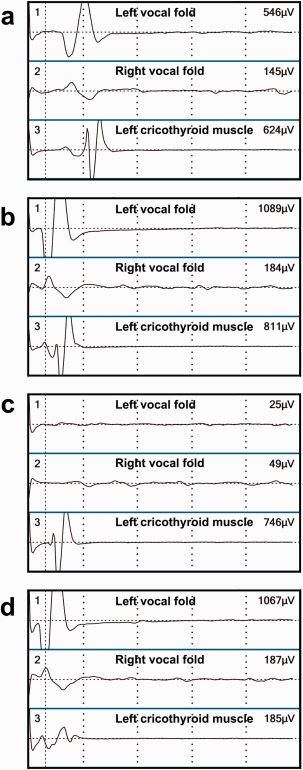
Electromyograms of the bilateral glottis muscles and the left cricothyroid muscle for patient 3, a 62‐year‐old man. (a) The left vagus was stimulated. (b) The recurrent laryngeal nerve (RLN) proximal to the branch (A in Fig. 2) was stimulated. (c) The anterior branch of the RLN (C in Fig. 2) was stimulated. (d) The RLN distal to the branch (B in Fig. 2) was stimulated. [Color figure can be viewed in the online issue, which is available at www.laryngoscope.com.]

Electrical stimulation of the left vagus at 2 mA and the left RLN proximal to the branches (A in Fig. 2) at 1 mA evoked clear responses of the glottis muscles and the left CTM (Fig. 3a, b, respectively). Simulation of the anterior branch (C in Fig. 2) showed a response of the left CTM and no glottis response (Fig. 3c). This response disappeared during thyroidectomy, and the branch became untraceable while we were dissecting the thyroid lobe from the trachea. Stimulation of the RLN distal to the anterior branch (B in Fig. 2) showed a glottis response without response of the CTM (Fig. 3d).

These findings indicated that the anterior branch of the left RLN innervated the left CTM. A summary of the electromyographic studies of patient 2 is given in Table 1. The evoked amplitudes of the CTM at stimuli on the proximal part of the left RLN and the branch of the RLN were 811 μV and 746 μV, respectively, values that were 34% to 37% of the value 2,182 μV obtained following the stimulation on the EBSLN. The patient did not report any voice change postoperatively.

**Table 1:** Electromyographic Data for Patient 2, a 54‐Year‐Old Female.

Site of Stimulation	Evoked Response Amplitude, μV	
	Ipsilateral Vocal Fold	Ipsilateral CTM
Left EBSLN	57	2,182
Left vagus	546	624
Left RLN‐central	1,089	811
Branch of RLN	25	746
Left RLN‐peripheral	1,067	185

Evoked responses were recorded with a trachea tube with surface electrodes for the vocal folds and with a pair of needle electrodes for the CTM. The EBSLN and RLN were stimulated at 1 mA and the vagus at 2 mA.

CTM = cricothyroid muscle; EBSLN = external branch of the superior laryngeal nerve; RLN = recurrent laryngeal nerve; RLN‐central = the RLN central to the branch; RLN‐peripheral = the RLN peripheral to the branch.

### Patient 3

A 62‐year‐old man underwent a total thyroidectomy and central node dissection with a tracheal tube with surface electrodes for intraoperative nerve monitoring inserted between his vocal folds.^10^ Before electrical stimulation of the EBSLN, the vagus, and the RLN, a pair of needle electrodes was inserted into the pars recta of the ipsilateral CTM. Both EBSLNs were detected with electrical stimulation and visual contraction of the CTM, visually identified, and preserved. Electrical stimulation of the right RLN at 1 mA evoked visual contraction of the right CTM and a strong electromyographic response at the pair of needle electrodes inserted into the CTM, as well as a glottis response at the tracheal tube electrodes.

We found a very fine branch near Berry's ligament running anteriorly with a small vessel, which ran along the caudal edge of the right CTM. Simulation of the branch at 0.5 mA yielded clear visual contraction of the right CTM (see Supporting Video 2 in the online version of this article) and a clear wave of electromyography for the muscle. During the tracing of the branch and the dissection of the right thyroid lobe, the contraction response of the CTM was lost and the branch became untraceable. Repeated stimulation of the right RLN lost the CTM response in the presence of the glottis response.

Based on these findings, we concluded that the very fine branch innervated the CTM and the branch was paralyzed during the surgical procedure. Stimulation of the left RLN did not yield any response of the left CTM visually or electromyographically.

A summary of this patient's electromyographic studies is given in Table 2. The evoked amplitude of the CTM at stimulation on the right RLN was 2,433 μV, which was approximately 31% of the value 7,839 μV obtained following the stimulation of the right EBSLN. It is of note that the evoked responses for the CTM were obtained with a pair of electrodes inserted directly into the muscle, whereas the evoked responses for the vocal folds were obtained with surface electrodes placed between his vocal folds. Postoperatively, the patient did not notice any significant change in his voice.

**Table 2:** Electromyographic Data of Patient 3, a 62‐Year‐Old Male.

Site of Stimulation	Evoked Response Amplitude, μV	
	Ipsilateral Vocal Fold	Ipsilateral CTM
Right EBSLN	104	7,839
Right vagus	460	2,140
Right RLN	493	2,433
Left EBSLN	16	7,901
Left RLN	640	119

Evoked responses were recorded with a trachea tube with surface electrodes for the vocal folds and with a pair of needle electrodes for the CTM. The EBSLN and RLN were stimulated at 1 mA, and the vagus was stimulated at 2 mA.

CTM = cricothyroid muscle; EBSLN = external branch of the superior laryngeal nerve; RLN = recurrent laryngeal nerve.

### Patient 4

**Figure 4:**
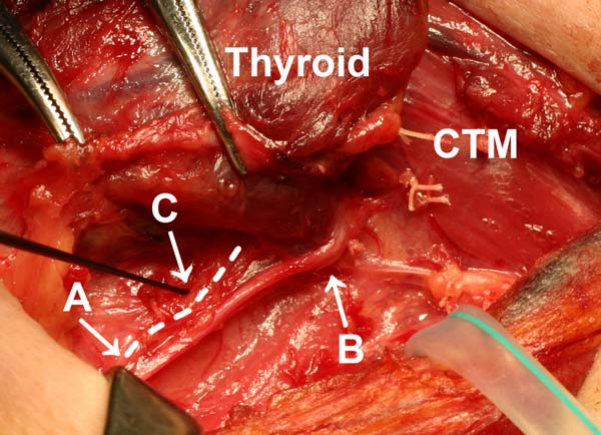
The left recurrent laryngeal nerve (RLN) and its branch in patient 4, a 44‐year‐old man. Electrical stimulation of the caudal potion of the RLN (A) evoked a twitch of the cricothyroid muscle (CTM) as well as a laryngeal twitch, whereas stimulation of the RLN near its laryngeal entry (B) showed a laryngeal twitch only. Careful observation found a very fine branch of the RLN (illustrated with a broken line). Stimulation of the branch (C) yielded a CTM twitch without a laryngeal twitch. The branch ran cranially and penetrated Berry's ligament, where it became untraceable. Head is to the right.

A 44‐year‐old man underwent a total thyroidectomy with central node dissection. On the right side, electrical stimulations at the vagus and at the caudal portion and the laryngeal entry point of the RLN showed a clear twitch of the CTM, and no branch of the RLN was found. On the left side, however, electrical stimulations at the vagus and at the caudal portion of the RLN showed a clear twitch of the CTM, whereas stimulation at the laryngeal entry of the RLN did not show CTM twitch. We thus carefully searched for and found a very fine branch of the RLN that ran anteriorly and cranially along the left RLN (Fig. 4). Electrical stimulation of the branch (C in Fig. 4) evoked clear visual contraction of the left CTM. Electrical stimulation of the RLN proximal to the branch (A in Fig. 4) also yielded a CTM twitch as well as a laryngeal twitch; however, stimulation of the RLN distal to the branch (B in Fig. 4) showed a laryngeal twitch only (see Supporting Video 3 in the online version of this article). We tried to trace the branch with the use of surgical loupes. It ran into Berry's ligament, where we lost the branch and the electrical response was lost.

None of the patients reported changes in their voices. Postoperative laryngoscopic examinations did not detect any abnormality, and postoperative phonation function tests in three of the patients preformed approximately 6 months after surgery showed no conclusive changes in frequency or voice level of their fundamental voices and high‐pitch voices and in the maximum phonation time compared to their preoperative values (Table 3). Patient 4 did not undergo a postoperative phonation function test because of the short period of time since his surgery.

**Table 3:** Phonation Function Data of the Four Patients With Papillary Thyroid Carcinoma, Before and After Surgery.

Patient	Study	Fundamental Voice		High‐Pitch Voice		MPT, s
	Preop/Postop, wk	Frequency, Hz	Voice Level, dB	Frequency, Hz	Voice Level, dB	
1	Preop	258	90	396	93	30
	26	247	80	811	105	20.3
2	Preop	267	86	703	92	33.4
	26	215	71	534	84	33.7
3	Preop	184	94	349	98	13.4
	26	181	92	718	98	14.1
4	Preop	119	87	570	93	16.4
	ND					

MPT = maximum phonation time; ND = not done because of a short postoperative period; Postop = after surgery; Preop = before surgery; wk = weeks after surgery.

## DISCUSSION

The present report on a selected and limited number of patients clearly demonstrated the presence of an inverse innervation of the CTM by extralaryngeal branches of the RLN in these patients. Contraction of the CTM by the electrical stimulation of the vagus and the RLN was visually observed in all four of the patients, and was confirmed with electromyographic studies with a pair of electrodes inserted into the pars recta of the CTM in two of the patients. The human communicating nerve^2^, ^3^, ^4^, ^5^ between the EBSLN and the RLN can conduct neural stimulation not only from the EBSLN to the glottis muscles^7^ but also inversely from the RLN to the CTM in the larynx.^7^, ^8^ Here we found and report that extralaryngeal branches of the RLN can also inversely innervate the CTM in some patients.

The physiological implications of this phenomenon of inverse innervation of the CTM by the RLN are not clear. Individuals with this aberrant innervation might have milder symptoms of paralysis of the EBSLN when the EBSLN is injured, because part of the CTM will remain innervated. Individuals with this reverse innervation might have greater changes in their voices when the RLN is injured, because part of the CTM will also become paralytic or weakened.

Patients with extralaryngeal branches of the RLN that innervate the CTM as shown in the present report could be expected to have a very high risk of paralysis of the branches, as was the cases in the present patients. The evoked amplitude of the CTM at stimulation of the RLN was 31% of the value obtained following the stimulation of the EBSLN in patient 2, and it was 37% in patient 3. Thus, these aberrant communications should have some significant clinical effects.

The extralaryngeal branches are very susceptible to injury during thyroidectomy, and their injury may be unavoidable, as was the case in the present patients. Injury of the EBSLN causes a lower and weaker voice and shorter maximum phonation time.^1^ Our patients, however, did not report any changes in their voices, and their phonation function tests did not show clear changes postoperatively. Because of the small number of patients and the low reliability of the test, the result of which depends on the patient's effort, we cannot make any conclusions regarding the influence of the injury of the branches. However, if they had professions involving their voices such as singing, they might have noticed changes in their voices.

These four patients were seen over a 27‐month period. During this period, 3,932 patients underwent thyroid surgeries as their initial operation at Kuma Hospital and 6,031 RLNs were exposed. Thus, the incidence of extralaryngeal branches innervating the CTM is calculated as 0.07% of RLNs. However, this study was not performed prospectively. All of the present four cases were found only by two surgeons (a.m. and h.m.), who both have a special interest in intraoperative neural monitoring. The actual incidence of the branches of the RLN innervating the CTM might be much higher. During the period, these two surgeons performed 740 initial thyroidectomies and exposed 1,194 RLNs. Thus, the true incidence might be >0.34%.

To avoid overlooking the branches, we would like to recommend the following procedures: 1) First, stimulate the vagus or the caudal portion of the RLN. 2) If a CTM twitch is obvious, stimulate the RLN near its laryngeal entry point. 3) If the latter is negative for a CTM twitch, search for possible branching of the RLN between these stimulation points of the RLN.

## CONCLUSION

We report here the presence of extralaryngeal branches of the RLN that inversely innervated the CTM, which has not been reported previously, to our knowledge. These patients carried a high risk of paralysis of these branches during thyroid surgery, possibly undergoing voice changes following thyroid surgery. The clinical implications and the incidence of this phenomenon remain to be clarified.

## Supporting information

Additional supporting information can be found in the online version of this article.

Supporting Information Video 1.

Supporting Information Video 2.

Supporting Information Video 3.
